# PCA driven mixed filter pruning for efficient convNets

**DOI:** 10.1371/journal.pone.0262386

**Published:** 2022-01-24

**Authors:** Waqas Ahmed, Shahab Ansari, Muhammad Hanif, Akhtar Khalil

**Affiliations:** 1 Faculty of Computer Science and Engineering, Ghulam Ishaq Khan Institute of Engineering Sciences and Technology Topi, Swabi, KPK, Pakistan; 2 iFhaja Research Consultation, Peshawar, KPK, Pakistan; Taipei Medical University, TAIWAN

## Abstract

Deployment of the deep neural networks (DNNs) on resource-constrained devices is a challenging task due to their limited memory and computational power. In most cases, the pruning techniques do not prune the DNNs to full extent and redundancy still exists in these models. Considering this, a mixed filter pruning approach based on principal component analysis (PCA) and geometric median is presented. First, a pre-trained model is analyzed by using PCA to identify the important filters for every layer. These important filters are then used to reconstruct the network with a fewer number of layers and a fewer number of filters per layer. A new network with optimized structure is constructed and trained on the data. Secondly, the trained model is then analyzed using geometric median as a base. The redundant filters are identified and removed which results in further compression of the network. Finally, the pruned model is fine tuned to regain the accuracy. Experiments on CIFAR-10, CIFAR-100 and ILSVRC 2017 datasets show that the proposed mixed pruning approach is feasible and can compress the network to greater extent without any significant loss to accuracy. With VGG-16 on CIFAR-10, the number of operations and parameters are reduced to 18.56× and 3.33×, respectively, with almost 1% loss in the accuracy. The compression rate for AlexNet on CIFAR-10 dataset is 2.61× and 4.85× in terms of number of operations and number of parameters, respectively, with a gain of 1.2% in the accuracy. On CIFAR-100, VGG-19 is compressed by 16.02 X in terms of number of operations and 36× in terms of number of parameters with a 2.6% loss of accuracy. Similarly, the compression rate for VGG-19 network on ILSVRC 2017 dataset is 1.87× and 2.4× for operations and parameters with 0.5% loss in accuracy.

## Introduction

Convolutional Neural Networks (CNNs) have achieved state of the art performance in many applications such as face recognition [1], object detection [2], semantic segmentation [3] and other classification tasks. Most of the CNN architectures are deeper and wider and contain a large number of parameters and operations which make them quite difficult to be deployed on power-constrained devices. In most of the cases, these networks are trained and deployed in computationally-rich devices such as graphics processing unit (GPU) for training and multi-core systems for deployment. However, these networks are also being deployed on mobile devices for certain applications such as voice and face recognition etc. In contrast to GPUs and multi-core systems, mobile devices have very limited memory and computational power. The modern deep neural networks are computationally expensive and memory intensive and require more computational power for deployment and training, it has become a challenge to bring the advances in neural network technology to mobile devices. Consequently, much work has been done in recent years, focused on reducing the size of pre-trained neural networks, making them capable to be deployed on mobile devices for inferences [4, 5]. The latest architectures such as inception module [6] or residual connection [7] have millions of parameters which require extensive computation and storage power. Table 1 shows the number of parameters (in millions) for some of the recently proposed CNN architectures. These architectures produce state of the art accuracy and most of the designers start with pre-trained networks for transfer learning purposes. These networks are rarely evaluated on the given datasets and only the classifier is trained and fine-tuned. This usually results in redundancy in the network. Therefore, it is of great importance to devise deep neural network models with relatively low complexity and high accuracy.

**Table 1:** Parameters overview of different CNN architectures.

S. No.	Network	Parameters (M)
1	VGG-19	143
2	VGG-16	138
3	AlexNet	61
4	SqueezeNet	1.24

**Fig 1:**
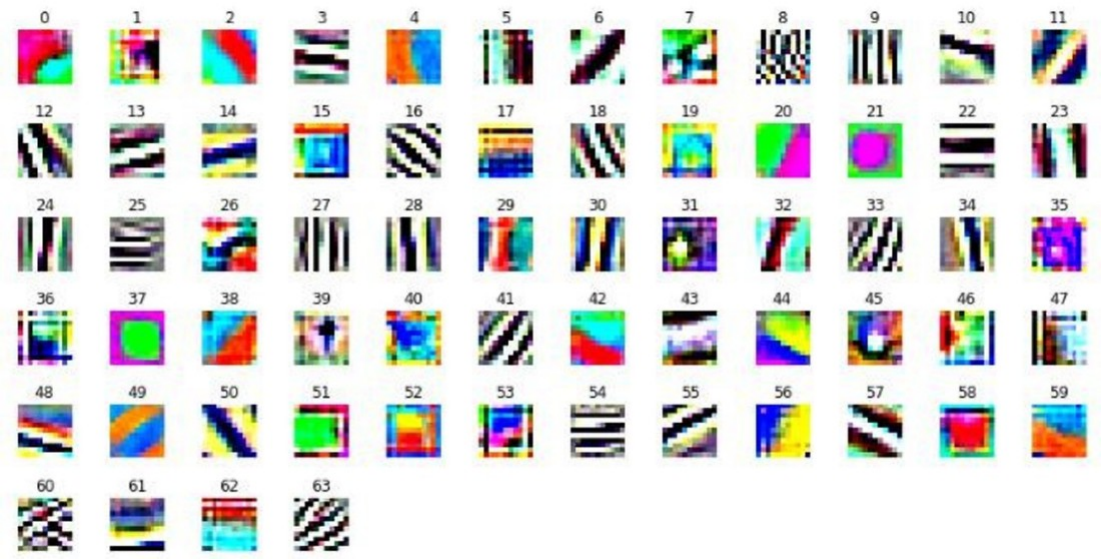
Visualization of first layer convolutional filters of AlexNet.

One of the major approaches for the optimization of CNNs is pruning. Pruning is an effective technique to reduce the size of the network by removing redundant filters or weights without effecting the accuracy [8–11]. It has been observed that the filters of well-established CNN models contain redundancy and removing these filters do not cause any degradation in the accuracy of the model. Fig 1 shows the filters of first convolutional layer of AlexNet trained on ImageNet dataset [12]. The redundancy in the filters is clearly visible.

Pruning can be classified into two categories, i.e. filter pruning [13, 14] and weight pruning [10, 15]. In weight pruning, the weight values which contribute less are directly deleted whereas a whole filter which contributes less is deleted in the filter pruning. Whether it is weight pruning or filter pruning, most of the pruning methods [16–18] involve multiple iterations to identify a suitable threshold for pruning. They start with a pre-trained network and compress the network layers one by one to find out pruning threshold for each layer. This process consumes heavy computational resources and time. Moreover, the network is not fully pruned by any of these approaches alone and redundancy still exists in the network. In order to achieve more pruned network, a mixed approach of filter pruning is presented in this paper where the focus is on redundancy rather than importance of filter towards accuracy. The work of [19] is utilized to compress the network both by reduction in number of layers and reduction in number of filters per layer by using the principal component analysis (PCA). The compressed network is then trained, and some redundant filters are pruned based on the approach given by [20].

The rest of the article is organized as follow. The overview of two step pruning method is given in Section 2. Recent work related to compression of neural networks is discussed in Section 3. In Section 4, the methodology of mixed filter pruning approach is discussed in detail. The results of the proposed scheme are discussed in Section 5. In Section 6, we conclude our work with some future recommendations.

## CNNs pruning

### Neural network compression using PCA

PCA is used for network analysis to get the compressed design having a fewer number of layers and a fewer number of filters in each layer without any retraining iterations [19]. A pre-trained network is selected and the activations of all layers are analyzed using PCA. The number of filters in each layer of the network is determined by the principal components required to explain 99.9% cumulative variance. The number of layers is determined based on when the number of filters in all layers start contracting. A new network with pre-determined number of convolutional layers and number of filters per layer is constructed and trained to get optimized network.

### Filter pruning via geometric median

**Fig 2:**
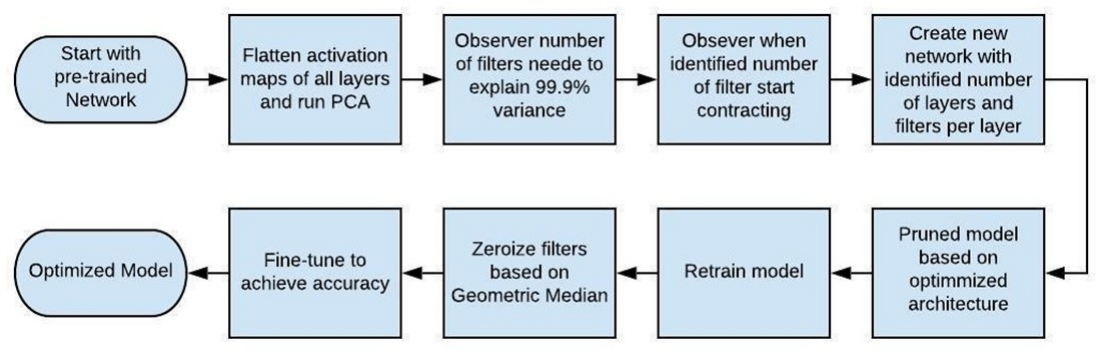
Flowchart of the proposed PCA driven mixed pruning technique.

As the centrality of data can be best expressed by geometric median, thus it can be utilized to find the filter which minimizes the summation of distances with other filters [20]. Geometric median is used to get common information of all the filters within a single layer. Then all the filters closed to geometric median are identified based on some threshold. As these filters can be represented by other filters in the layer, therefore pruning these has little impact on the network. These filters are then set to zero. The flow chart in Fig 2 shows the overall procedure of two step pruning of a convolutional neural network.

## Related work

In recent years, a number of CNN models are represented having a large number of parameters and memory cost. These models have yield state of the art performance in many applications [21]. However, one of the major drawbacks of these models is over-parameterization which results in heavy computational costs. To tackle this problem, various techniques are proposed to get more robust but computationally efficient models. The work on the acceleration and optimization of CNNs can be divided into four main categories [22], i.e. knowledge distilling, compact convolutional filters, low rank factorization and quantization and pruning. In pruning, multiple techniques were developed to filter out and remove the redundant filters or weights. Some of the main techniques are discussed below.

### Weight pruning

In this type of pruning techniques, the insignificant weights are identified using a predefined criteria and pruned. In [8], a deep compression technique is proposed containing three stages. They first pruned the network while retaining only important weights, then weight sharing was enforced by quantization methods and finally Huffman coding was applied to further reduce the size of the network. They successfully reduced the size of the VGG16 by 49×, however this method requires specialized hardware due to the formation of unstructured sparsity [23].

In [9], the authors suggested an approach for energy-efficient implementation of hardware for large scale neural networks. They have converted a neural network into an approximate neural network by analyzing the importance of a neuron. This was done by using the back propagation mechanism and then subsequently removing the least important connections. This technique is a good approach towards network compression however it requires repeated iterations and consumes huge amount of resources. Similarly, [16, 24] have implemented the weight pruning techniques in a similar manner which results in unstructured sparsity and requires repeated iteration to identify the weights that are less important.

### Filter pruning

In this technique, the entire filter is removed if found insignificant towards the final accuracy of the network. In [25], authors have proposed a scheme to prune the convolutional kernels in neural networks by interleaving criteria-based pruning with fine tuning. They have approximated the changes in the cost function of network parameters by using Taylor expansion. The authors in [11] have proposed the method to prune the redundant filters and retrain the accuracy. They have used the L1-norm to estimate the importance of the filter and filtered out the unimportant filters thus leaving no unstructured sparsity in the pruned network. In [10], authors have removed the entire insignificant filters along with their feature maps to reduce the computational costs significantly. Their work is a good attempt to compress the network and they acheived up to 34× reduction in FLOPs for VGG16 on CIFAR10. Similarly, authors in [26] used the statistics of the next layer to prune the filters in the current layer. These methods do require iterative retraining which consumes heavy computational resources and time. In [25], authors have suggested analysis of all the layers together, however it too requires many repetitive iterations.

In [27], authors have removed the filters by introducing multiple losses to each layer. They selected the least important filters by using these losses and the process continues until a stopping criterion meets. This process is done for all layers and it takes multiple iterations.

Recently [28] proposed a filter pruning technique based on maximizing the number of zeroes in filters and eliminating the filters having more non-zero parameters. Their idea is based on the fact that if there is any zero value out of two inputs to a multiplier then multiplication operation can be skipped. They are successful in replacing 82% of multiplications with zero-skip multiplications that do not switching energy in multiplication circuits. However, zero-skip multiplication operations are still performed and are not completely eliminated.

### Weight matrices approximation

The third category of techniques to reduce the size and to accelerate the network is the approximation of weight matrices [29–31]. This is done either by quantization or low rank matrix approximation methods. In [32] and [18], authors have used techniques of quantization to reduce the size of the network. Quantization also involves multiple iteration which becomes a bottleneck to large networks. The authors in [33] have proposed to train the CNNs with binary weights and activations. They have divided the network into segments and trained all the segments in a sequential way. The idea of dividing the network into segments is due to the fact that a group can be reconstructed by combining the set of similar binary branches. However, this technique requires custom training methods. In [17], authors have proposed a similar technique to reconstruct the network having fewer parameters however the compression is mainly achieved by regularization. One of the major drawbacks of the regularization process is that it adds more hyper parameters into the process which takes more iterations to achieve optimized model.

### Learning sparsity patterns

The techniques in this category of pruning usually modify the loss function for the model optimization [14]. The weights are detected that minimize the loss while satisfying the pruning criterion. In [34], authors have proposed a method to prune the network during training by using *L*_0_-norm regularization in neural networks. The scheme proposed to set the identified weights to zero by including a collection of non-negative stochastic gates. These methods do not guarantee structured sparsity in the network and takes longer training time. The authors in [35] have presented a non-structured architecture that contains a nucleus of connections at the start and the connections are optimized during the training process. This method requires more computational resources to optimize the network.

Apart from these four pruning categories, there are some methods that combine different techniques together. In [36], authors have combined weight pruning along with filter pruning to prune the network to the maximum extent. They have used information from the next layer to prune the filters in the current layer. But their method requires a huge amount of computation; first for identifying the insignificant filters and second for the fine tuning to regain accuracy.

## Mixed filter pruning

Most of the pruning methods involve multiple iterations to identify a suitable threshold for pruning. They start with a pre-trained network and compress the network’s layers one by one to find out pruning threshold for each layer. This process consumes heavy computational resources and time. Moreover, the network is not fully pruned by any of these approaches alone and redundancy still exists in the network. In order to achieve a more optimized network, a 2-step technique of filter pruning is presented in this section.

First, PCA is used to analyze the network to get the compressed design having fewer number of layers and fewer number of filters in each layer without any retraining iterations. A pre-trained network is taken, and the activations of all layers are analyzed using PCA. The number of filters in each layer of the network is determined by the principal components required to explain 99.9% cumulative explained variance. The number of layers are determined based on contracting of filters in all layers. A new network with determined number of convolutional layers and number of filters per layer is constructed and trained to get optimized network.

Second, geometric median is used to get common information of all the filters within a single layer. Then all the filters are identified which are nearest to geometric median in that layer, based on some threshold. These filters can be represented by other filters in the layer, hence pruning them has little impact on the network. These filters are then removed from the network.

### Step1: Pruning filters via PCA

PCA is used to analyze a pre-trained network without any retraining iterations to get an optimized design. The redundancy in the network is minimized in terms of width (number of filters per layer) and depth (number of layers). The PCA analysis is performed on a pre-trained network by analyzing activations of all the layers simultaneously. The width of the network is determined by the number of principal components required to explain 99.9% cumulative explained variance. Depth of the network is determined based on when the width of the layers starts contracting.

In complex data, difficulty in visualization and performing computations on data increases with the increase in the data dimensions. PCA is an analysis technique for the complex data in which only the significant dimensions are retained and the redundant dimensions are removed. For better understanding of the PCA, following terms are defined.

Variance is the measurement of variability in the data or how far the data points are with respect to each other. It can be computed as the average squared deviation from the mean as

$$\begin{array}{c} var\left(x\right)=\frac{\sum {\left(x_{i}-\bar{x}\right)}^{2}}{N} \end{array}$$ (1)

Where *x*_*i*_ is the value of one observation *x* in the *i*^*th*^ dimension, $\bar{x}$ is the mean value of all observations and *N* is the total number of observations.

Covariance measures the degree to which corresponding elements from two ordered datasets are moving in the same direction. Positive covariance of two variables x and y indicates that x increases with the increase in y. Negative covariance indicates x in deceasing with the increase in y. Zero covariance indicates that both variables are not related. Covariance can be computed as

$$\begin{array}{c} cov\left(x,y\right)=\frac{\sum \left(x_{i}-\bar{x}\right)\left(y_{i}-\bar{y}\right)}{N} \end{array}$$ (2)

xi is the value of x in ith dimension Where *x*_*i*_ and *y*_*i*_ represent the value of variable *x* and *y* in the *i*^*th*^ dimension; respectively. The $\bar{x}$ and $\bar{y}$ are the mean values of *x* and *y*; respectively. *N* is the total number of values.

For a matrix *A*, an eigenvector is represented by *x* such that on the multiplication of *A* and *x* the direction of the resultant vector is the same as vector *x* scaled by λ. Mathematically,

$$\begin{array}{c} Ax=\lambda x \end{array}$$ (3)

Here, *A* is an arbitrary matrix, λ is an eigenvalue of *A* and *x* is the eigenvector corresponding to the eigenvalue.

A covariance matrix for a dataset having four-dimension a, b, c and d is shown as

$$\begin{array}{c} C=[\begin{array}{cccc} V_{a} & C_{a,b} & C_{a,c} & C_{a,d}\\ C_{a,b} & V_{b} & C_{b,c} & C_{b,d}\\ V_{a,c} & C_{b,c} & V_{c} & C_{c,d}\\ V_{a,d} & C_{b,d} & C_{c,d} & V_{d} \end{array}] \end{array}$$ (4)

Where *V*_*a*_ represents variance along the dimension a and *C*_*a*,*b*_ represents covariance along the dimensions a and b. For a matrix *X* having *m* × *n* dimensions where *n* is the number of data points having *m* dimension, the covariance matrix can be calculated as

$$\begin{array}{c} C_{x}=\frac{\left(X-\bar{X}\right){\left(X-\bar{X}\right)}^{T}}{n-1} \end{array}$$ (5)

Following are the major steps involved in computation of PCA:

1. Calculation of the covariance matrix *X* for the given data points.
2. Calculation of eigenvectors and corresponding eigenvalues.
3. Sorting of eigenvectors based on their eigenvalues in decreasing order.
4. For a certain threshold *k*, choose first *k* eigenvectors which are the new *k* dimensions.
5. Transformation of the original *m* dimensional data points into new *k* dimension.

PCA tries to retain high variance along the dimensions, i.e. data to be spread out by removing the correlated dimension. There are two major goals of PCA: (1) to find out linearly independent dimensions so that the data can be represented without any loss, and (2) the original dimension can be reconstructed by those linearly independent dimensions. Once the covariance matrix of the original dataset is calculated, our goal is to transform the original data points so the covariance matrix of the new transformed data points become diagonal matrix. For the *m* dimensional *n* data points, the size of covariance matrix is

$$\begin{array}{c} X=m\times n \end{array}$$ (6)

The *k* principal dimensions are chosen with respect to the *k* largest eigenvalues. Thus

$$\begin{array}{c} P=k\times m \end{array}$$ (7)

Then input data in reduced dimensions are given as

$$\begin{array}{c} Y=PX \end{array}$$ (8)

That is,

$$\begin{array}{c} \left(k\times n)=(k\times m)\times (m\times n\right) \end{array}$$ (9)

This shows *n* data points having *k* dimensions. In other words,

$$\begin{array}{c} {\left[new\;data\right]}_{k\times n}={\left[top\;k\;eigenvectors\right]}_{k\times m}\times {\left[original\;data\right]}_{m\times n} \end{array}$$ (10)

#### Input data to PCA

It is observed that the filters present in the layers of deep convolutional neural networks are highly correlated and contains redundancy. These filters might be detecting the same feature hence making no contribution towards the network performance. In order to reduce the redundancy present in the CNN, activations are used as feature values of the filters. The feature value of a filter is calculated by its convolution with the input patch. The input to PCA is a two-dimensional matrix representing the input sample as its row and corresponding feature value as its column. In the input matrix to PCA, a data point at the location [*i*, *j*] is the activation generated by the convolution of *i*^th^ input patch with *j*^th^ filter. This input patch convolves with all the filter making a full row of features values.

**Fig 3:**
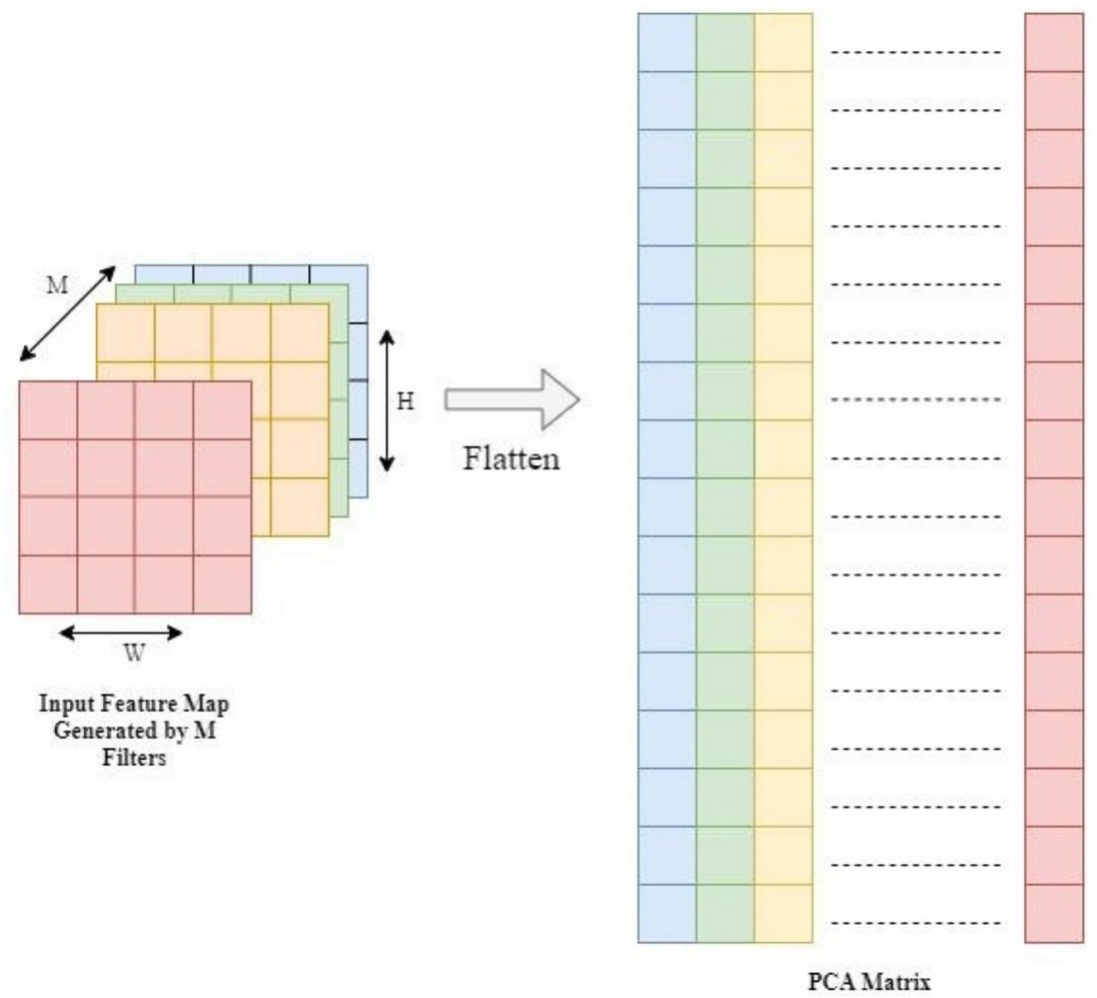
Flattened activation matrix as input to PCA.

Let *A*_*L*_ be the matrix that is obtained by the activations of layer *L* in a forward pass. The first input patch having size equal to the kernel size convolves with the kernel to produce first pixel of the output activation map. This input patch convolves with *M* filters to form a row in the output map having dimension *R*^1×1×*M*^. The next input patch is also convolved with all the filters to form another row in the output activation map. The resultant activation map will be having the dimension *A*_*L*_ *ϵR*^*N* × *H* × *W* × *M*^, where *N* is the batch size, *H* is the height of activation map and *W* is its width. We can flatten this matrix as

$$\begin{array}{c} A_{L}\epsilon R^{N\times H\times W\times M}\to B_{L}\epsilon R^{D\times M} \end{array}$$ (11)

where *D* = *N* × *H* × *W*. The input matrix to the PCA is described in the Fig 3.

The flattening of the matrix results into the collection of *N* × *H* × *W* samples in one forward pass. PCA is then performed on *B*_*L*_. Singular Value Decomposition (SVD) is performed on mean normalized symmetric matrix to get *M* number of eigenvectors *V*_*i*_ and eigenvalues λ_*i*_. Total variance *T* is the sum of diagonal elements in the covariance matrix and is equal to the sum of individual variance of the parameters. This is known as trace and is represented as $tr({B_{L}}^{T}B_{L})$. As trace *AB* = trace *BA*, hence trace *S* = trace *W*(*ΛWT*) = trace (*ΛWT*)*W* = trace *Λ*, i.e. trace of *B*_*L*_ is sum of its eigenvalues

$$\begin{array}{c} tr\left({B_{L}}^{T}B_{L}\right)=\sum_{i=1}^{M}\alpha_{ii}^{2} \end{array}$$ (12)

Or

$$\begin{array}{c} tr\left({B_{L}}^{T}B_{L}\right)=\sum_{i=1}^{M}\lambda_{i} \end{array}$$ (13)

Here each eigenvalue is supposed to be explaining λ_*i*_/*T* ratio of total variance. As all the eigenvalues are ordered in decreasing order, we can calculate how many cumulative eigenvalues are required to explain 99.9% of the explained total variance. These eigenvalues represent the significant dimensions in the layer and denoted by *S*_*L*_ which is given as

$$\begin{array}{c} S_{L}=\frac{\sum_{i=1}^{\hat{M}}\lambda_{i}}{\sum_{i=1}^{M}\lambda_{i}}=0.999 \end{array}$$ (14)

The $\hat{M}$ significant dimensions are helpful in optimizing the depth and width of the network.

#### Optimizing width and depth of the network

The significant dimensions of the filter space is determined as the number of uncorrelated filters that are able to define 99.9% variance. As discussed above, these PCA matrices are flattened and analyzed by PCA to define the significant dimensions for each layer. These dimensions determine the number of filters per layer in the network. If we consider every layer as a transformation function, in which the expansion of input data into higher dimensions is performed until the data become linearly separable, it implies that the width of the network per layer must be a non-decreasing function. The results show that the number of significant dimension (width of the layer) per layer increase up to certain layer and then starts decreasing. This implies that the layer with lower number of significant dimensions than the preceding layer is contributing no useful transformation towards the final results.

### Step2: Pruning filters via geometric median

Geometric median represents the central tendency in higher dimensions. We can compute the central tendency via geometric median as follow [37]. For a set of *n* points *a*_1_, *a*_2_, …, *a*_*n*_ where every *a*_*i*_ *ϵR*^*d*^, find a point *xϵR*^*d*^ that minimizes the sum of Euclidian distance to all the points.

$$\begin{array}{c} x=arg\;\underset{x}{\text{min}}f\left(x\right) \end{array}$$ (15)

where

$$\begin{array}{c} f\left(x\right)=\sum_{i=1}^{n}\left|x-a_{i}\right| \end{array}$$ (16)

For a particular layer *L*, geometric median can be used to get the common information pertaining to all the filters of *L*.

$$\begin{array}{c} x^{GM}=arg\;\underset{x}{\text{min}}\sum_{\overset{´}{j}=1}^{N_{j}+1}{\left|x-F_{i,\overset{´}{j}}\right|}^{2} \end{array}$$ (17)

where *xϵR*^*N*_*i*_ × *K* × *K*^.

After finding the geometric median, we can find the filter nearest to geometric median in that layer.

$$\begin{array}{c} F_{i,j^{*}}=arg\;\underset{F_{i,\overset{´}{j}}}{\text{min}}{\left|F_{i,\overset{´}{j}}-x^{GM}\right|}^{2} \end{array}$$ (18)

where $\overset{´}{j}\in \left[1,N_{i+1}\right]$.

The filters represented by *F*(*i*;j*) are the redundant filters and can be pruned out without any negative effect towards networks output. Now we can determine which filter in the ith layer has the minimum summation of distances with other filters.

$$\begin{array}{c} F_{i,x^{*}}=arg\;\underset{x}{\text{min}}\sum_{\overset{´}{j}\epsilon \left[1,N_{i+1}\right]}{\left|x-F_{i,\overset{´}{j}}\right|}^{2} \end{array}$$ (19)

where *xϵ*[*F*_*i*,1_, …, *F*_*i*,*N*+ 1_].

Here, the filters represented by *F*(*i*, x*) are those filters which share the most common information which means that their information can be replaced by other filters. The effect of removing these filters will be negligible and network can regain the accuracy with fine tuning.

## Results and discussion

The PCA driven mixed filter pruning approach was tested on three publicly available datasets and three neural networks. The CIFAR-10 and CIFAR-100 are online available on https://www.cs.toronto.edu/kriz/cifar.htmland ILSVRC2017 is available on https://image-net.org/challenges/LSVRC/2017/. The CIFAR-10 is tested on VGG-16 and AlexNet while CIFAR-100 and ILSVRC2017 are tested on VGG-19. Mixed pruning is applied on trained networks and redundant filters are pruned out. The compressed models are fine tuned for some epochs to regain the accuracy. On the given datasets and networks, mixed pruning has achieved state of the art results. The models are compressed up to 4.85× in number of parameters and 18.56× in operations without any significant loss in accuracy up to 2.65%.

### Methods

PCA driven mixed pruning approach is implemented using PyTocrh [38] library and was trained on NVIDIA Tesla K80 GPU. The training scheme consists of four different levels:

1. Training the network on given data without any pruning.
2. Training with pruning in terms of depth and width contraction of network based on the approach given by [19].
3. Training with pruning approach given by [20].
4. Training with filter pruning based mixed approach.

The specifications of the networks and datasets used in the experiments are described in Table 2.

**Table 2:** Networks and datasets used in the experiments.

Sr. No.	Network				Dataset			
	Name	Conv Layers	FC Layers	Parameters	Name	Classes	Train Images	Test Images
1	AlexNet	5	3	60	CIFAR-10	10	50,000	10,000
2	VGG-16	13	3	138	CIFAR-100	100	50,000	10,000
3	VGG-19	16	3	143	ILSVRC2017	1000	1 Million	150,000

### VGG-16 on CIFAR-10

**Fig 4:**
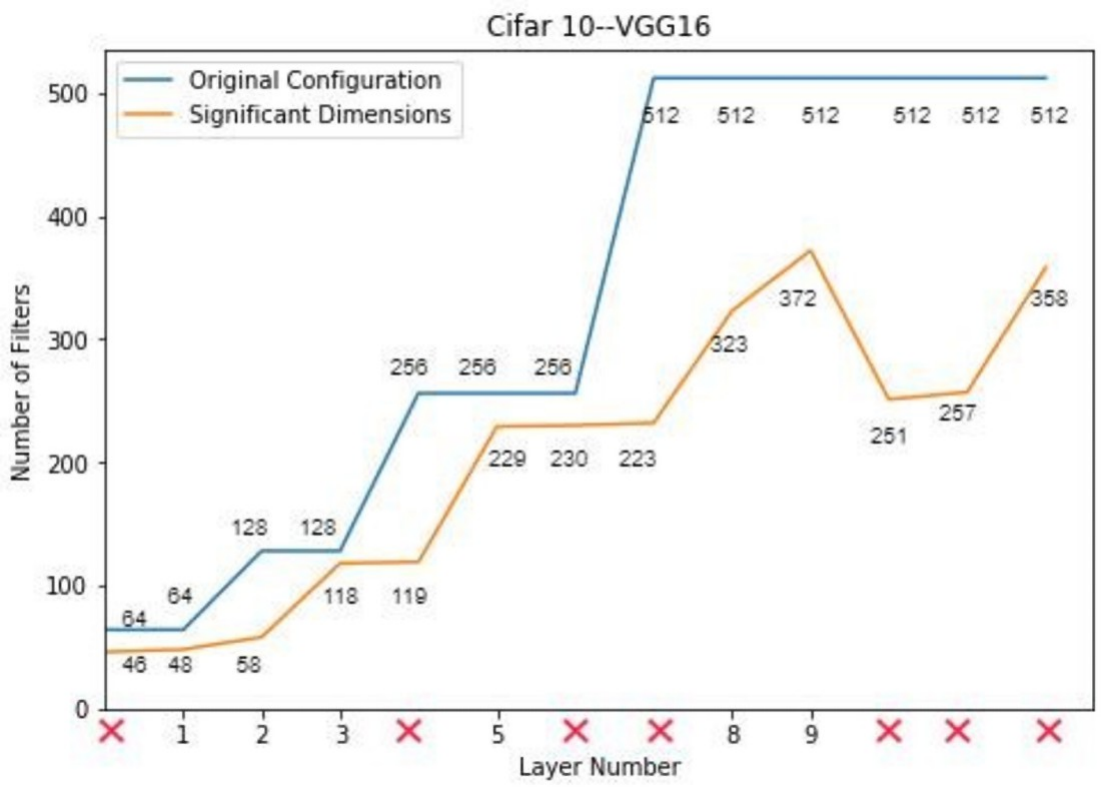
Compressed architecture of VGG-16 on CIFAR-10.

The VGG-16 network with batch normalization was trained on CIFAR-10. The activations of a pre-trained VGG-16 on CIFAR-10 were given to PCA as input to detect the significant dimensions. PCA is used as a dimensionality reduction tool. The significant dimensions represent the filters in a convolutional layer that contributes significantly towards the output. The significant dimension for the VGG-16 network on CIFAR-10 dataset is summarized in Table 3. Based on these significant dimensions, the depth of the network is also compressed along with reduction in number of filters per layer. This phenomenon is shown in the Fig 4. It has been observed that the significant dimensions of the network expand up-to certain limit and then starts contracting. Therefore, it can be concluded that the layer having less or same number of filters than the preceding layer do not contribute towards any significant transformation of input data. In this way, the redundant layers are removed from the network. The final network is compressed to six layers only.

**Table 3:** Configuration of VGG-16 on CIFAR-10.

Sr. No.	Type	Configuration
1	Initial Configuration	[64, 64, M, 128, 128, M, 256, 256, 256, M, 512, 512, 512, M, 512, 512, 512]
2	Significant Dimensions	[46, 48, M, 58, 118, M, 119, 229, 230, M, 223, 323, 372, M, 251, 257, 358]
3	PCA-Based Configuration	[48, 58, M, 118, 229, M, 323, 372]
4	Final Configuration	[30, 36, M, 72, 139, M, 165, 224]

After obtaining the optimized architecture, the network is initialized randomly and trained. The trained network is pruned again based on geometric median. In this approach, the redundant filters are detected and removed. With a little fine tuning of 50 epochs, the network regained its accuracy. The final number of parameters and operations of the network are reduced by 3.33 X and 18.56 X respectively. This is shown on Table 4.

**Table 4:** Comparison of VGG-16 on CIFAR-10.

Model	Accuracy	FLOPs	Parameters
Original	93.57%	1×	1×
PCA-Based [19]	93.36%	7.78×	2.82×
FPGM [20]	93.23%	1.42×	1.62×
FPZ [28]	92.63%	5.55×	2.12×
Proposed	92.38%	18.56×	3.33×

### AlexNet on CIFAR-10

AlexNet is a smaller CNN architecture compared to VGG style networks and it is not much over-parametrized. For the analysis of redundancy in the network, the activations obtained by convolution of input patches with the filters of every layer are given to PCA. As a dimensionality reduction tool, PCA is used to identify the significant elements from the input matrix which we call significant dimensions. These significant dimensions are then used to reconstruct the network based on the predetermined length and width. The elements of the network other than significant dimensions are considered redundant and can be discarded. By experiments, it has been observed that the redundancy in the newly constructed network based on the significant dimensions is still present. In order to prune the network further, geometric median based pruning technique is applied which further zeroized the redundant filters. The initial configuration, its significant dimensions and final configuration is shown in Table 5.

**Table 5:** Configuration of AlexNet on CIFAR-10.

Sr. No.	Type –	Configuration
1	Initial Configuration	[64, 192, 384, 256, 256]
2	Significant Dimensions	[58, 164, 329, 242, 200]
3	PCA-Based Configuration	[58, 164, 329, 242]
4	Final Configuration	[36, 99, 199, 146]

**Fig 5:**
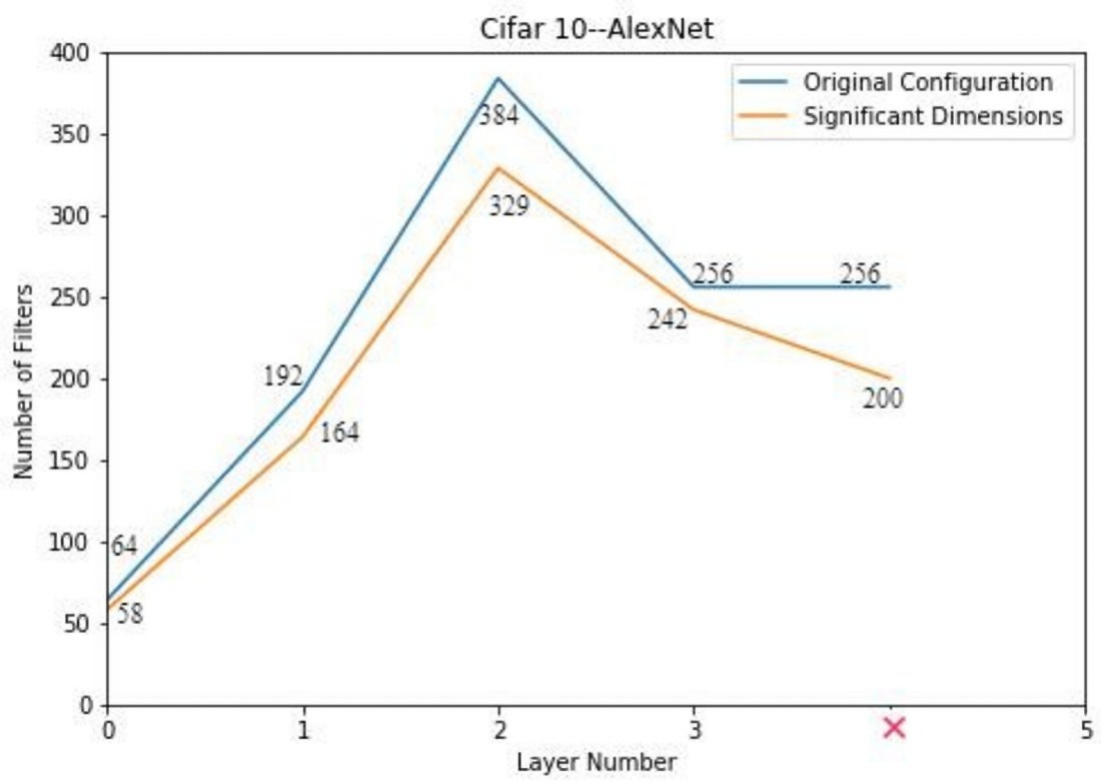
Compressed architecture of AlexNet on CIFAR 10.

The layer wise significant dimensions are the key factor to determine the length of the network. It can be concluded that the layer having less or same number of filters than the preceding layer do not contribute towards any significant transformation of input data. In this way, the redundant layers are removed from the network. The final network contains four layers. This is shown in Fig 5. The network is initialized randomly based on the compressed architecture obtained from PCA. This network is trained with the accuracy of 85.64% and the trained model is pruned again based on geometric median. Number of parameters in the final model are reduced by 4.85× and operations are reduced by 2.61×. The results are summarized in Table 6.

**Table 6:** Comparison of AlexNet on CIFAR-10.

Model	Accuracy	FLOPs	Parameters
Original	86.31%	1×	1×
PCA-Based [19]	86.64%	1.69×	4.33×
FPGM [20]	85.34%	2.35×	1.03×
Proposed	87.51%	2.61×	4.85×

### VGG-19 on CIFAR-100

VGG-19 is a deep convolutional neural network with 16 convolutional layers. This network is composed of sixteen convolutional layers, five pooling layers and three fully connected layers. VGG-19 is comparatively deeper network and it contains more redundancy. There might be a number of filters detecting the same feature and hence making no significant contribution towards the final out-put of the network. Therefore, this network is much overparametrized and can be compressed significantly. For the compression of this model, two step pruning method is proved to be effective. First, the activations of a pre-trained model are given to PCA for the analysis of significant dimensions. The redundant filters are identified by PCA by analyzing the activation maps of a pre-trained VGG-19 model.

**Fig 6:**
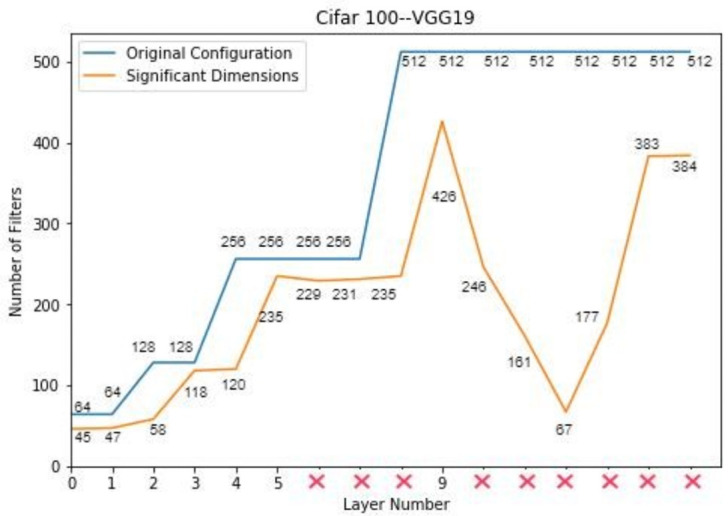
Compressed architecture of VGG-19 on CIFAR 100.

The analysis shows that the significant dimensions obtained by PCA are fluctuating across the length of the network. Those layers which have fewer or equal numbers of filters than their preceding layers are treated as insignificant and can be removed from the network as shown in Fig 6.

Once the network is compressed according to the significant dimensions identified by PCA, it is initialized randomly and trained again on the same dataset. Once the required accuracy is obtained, the network is analyzed again for redundancy by using geometric median. To eliminate the redundant filters, filter pruning via geometric median is applied on the trained network and some unimportant filters are set to zero. The final configuration of the network is shown in Table 7.

**Table 7:** Configuration of VGG-19 on CIFAR-100.

Sr. No.	Type	Configuration
1	Initial Configuration	[64, 64, 128, 128, 256, 256, 256, 256, 512, 512, 512, 512, 512, 512, 512, 512]
2	Significant Dimensions	[46, 47, 58, 118, 120, 235, 229, 231, 235, 426, 246, 161, 67, 177, 383, 384]
3	PCA-Based Configuration	[45, 47, 58, 118, 120, 235, 426]
4	Final Configuration	[28, 29, 36, 72, 72, 142, 257]

The results show significant improvement in the network efficiency in terms of number of operations and parameters without any significant loss in accuracy. The FLOPs are reduced by 16.02× and parameters are reduced by 36.4× with a total loss of 2.65% in accuracy. The results are shown in Table 8.

**Table 8:** Comparison of VGG-19 on CIFAR-100.

Model	Accuracy	FLOPs	Parameters
Original	70.42%	1×	1×
PCA-Based [19]	69.88%	14.41×	7.91×
FPGM [20]	69.21%	1.11×	2.66×
Proposed	67.77%	16.02×	36.42×

### VGG-19 on ILSVRC2017

The final experiment was performed on ILSVRC2017 dataset with batch normalized VGG-19 network. By using the two-step pruning methods, the activations of a pretrained model are given to PCA for the analysis of significant dimensions. The redundant filters are identified by PCA by analyzing the activation maps of a pretrained VGG-19 model.

The compressed architecture on the network is analyzed again for redundancy by using geometric median and the redundant filters are zeroized. The summary for the filters in different stages is given in Table 9.

**Table 9:** Configuration of VGG-19 on ILSVRC2017.

Sr. No.	Type	Configuration
1	Initial Configuration	[64, 64, 128, 128, 256, 256, 256, 256, 512, 512, 512, 512, 512, 512, 512, 512]
2	Significant Dimensions	[6, 30, 49, 100, 169, 189, 205, 210, 400, 455, 480, 490, 492, 492, 492, 492]
3	PCA-Based Configuration	[6, 30, 49, 100, 169, 189, 205, 210, 400, 455, 480, 490, 492, 492, 492, 492]
4	Final Configuration	[5, 18, 31, 30, 103, 115, 124, 126, 240, 277, 288, 294, 296, 296, 296, 296]

The compression in terms of number of parameters and number of operations is not as much significant as in case of CIFAR-100 dataset. This is because ILSVRC2017 is a huge dataset and most of the filters tend to learn some features. The Table 10 shows the results of this experiment.

**Table 10:** Comparison of VGG-19 on ILSVRC2017.

Model	Accuracy	FLOPs	Parameters
Original	74.24%	1×	1×
PCA-Based [19]	74%	1.3×	1.14×
FPGM [20]	73.97%	1.42×	1.27×
Proposed	73.69%	1.87×	2.4×

## Conclusion

Deep neural networks have achieved high performance in different data intensive applications. However, their performance comes with a cost of high computational requirement. One possible solution is to compres the network using pruning. Most of the filter pruning techniques are iterative and consumes huge amount of time and computation to compress the network. These techniques have achieved good results however it is observed that the redundancy still exist in the networks and the networks are not fully pruned. A mixed pruning method without the involvement of any repetitive iterations is presented in this paper to remove the redundancy in the convolutional neural networks. First, the trained model is analyzed using PCA and its significant filters are detected by giving the activations as input to PCA. Based on these dimensions, a new network is initialized randomly with a smaller number of layers and smaller number of filters per layer. The newly formed model is trained and analyzed again for redundancy using geometric median. The identified redundant filters are then set to zero. The network is then fine tuned to regain its accuracy with an optimized number of layers.

The proposed network pruning method helps significantly in reducing the computational cost of the network but at the same also cause a minimal loss in accuracy. One of the possible future direction may be working on the pruning with improved accuracy.
